# Bioreactor Scalability: Laboratory-Scale Bioreactor Design Influences Performance, Ecology, and Community Physiology in Expanded Granular Sludge Bed Bioreactors

**DOI:** 10.3389/fmicb.2017.00664

**Published:** 2017-05-01

**Authors:** Stephanie Connelly, Seung G. Shin, Robert J. Dillon, Umer Z. Ijaz, Christopher Quince, William T. Sloan, Gavin Collins

**Affiliations:** ^1^Infrastructure and Environment, School of Engineering, University of GlasgowGlasgow, UK; ^2^School of Environmental Science and Engineering, Pohang University of Science and TechnologyPohang, South Korea; ^3^Microbial Communities Laboratory, National University of Ireland GalwayGalway, Ireland; ^4^Microbiology and Infection, University of WarwickWarwick, UK

**Keywords:** 16S rRNA gene, anaerobic digestion, EGSB, Illumina MiSeq, laboratory-scale, full-scale, industrial wastewater, specific methanogenic activity

## Abstract

Studies investigating the feasibility of new, or improved, biotechnologies, such as wastewater treatment digesters, inevitably start with laboratory-scale trials. However, it is rarely determined whether laboratory-scale results reflect full-scale performance or microbial ecology. The Expanded Granular Sludge Bed (EGSB) bioreactor, which is a high-rate anaerobic digester configuration, was used as a model to address that knowledge gap in this study. Two laboratory-scale idealizations of the EGSB—a one-dimensional and a three- dimensional scale-down of a full-scale design—were built and operated in triplicate under near-identical conditions to a full-scale EGSB. The laboratory-scale bioreactors were seeded using biomass obtained from the full-scale bioreactor, and, spent water from the distillation of whisky from maize was applied as substrate at both scales. Over 70 days, bioreactor performance, microbial ecology, and microbial community physiology were monitored at various depths in the sludge-beds using 16S rRNA gene sequencing (V4 region), specific methanogenic activity (SMA) assays, and a range of physical and chemical monitoring methods. SMA assays indicated dominance of the hydrogenotrophic pathway at full-scale whilst a more balanced activity profile developed during the laboratory-scale trials. At each scale, *Methanobacterium* was the dominant methanogenic genus present. Bioreactor performance overall was better at laboratory-scale than full-scale. We observed that bioreactor design at laboratory-scale significantly influenced spatial distribution of microbial community physiology and taxonomy in the bioreactor sludge-bed, with 1-D bioreactor types promoting stratification of each. In the 1-D laboratory bioreactors, increased abundance of *Firmicutes* was associated with both granule position in the sludge bed and increased activity against acetate and ethanol as substrates. We further observed that stratification in the sludge-bed in 1-D laboratory-scale bioreactors was associated with increased richness in the underlying microbial community at species (OTU) level and improved overall performance.

## Introduction

Anaerobic digestion (AD) is a microbially-driven wastewater treatment process enabling energy, nutrient and water recovery from wastes. The development of new biotechnologies such as those used for the AD of wastes, has historically followed the empirical route from laboratory-scale through to pilot- and full-scale trials (Switzenbaum, 1995; Tchobanoglous et al., 2004; O'Flaherty et al., 2006; Shida et al., 2012). Advantages of testing and development at laboratory-scale prior to scale-up include greatly reduced capital and construction costs, rapid project turnaround and minimal effluent generation, which collectively provide flexibility to test hypotheses and optimize processes. However, whilst many parameters may be readily reproduced across scales, e.g., operating temperature and hydraulic retention times, others, such as bioreactor geometry or hydrodynamics, may not. Compounding this, published research rarely, if ever, tracks the success of environmental biotechnology trials across each of the laboratory-, pilot-, and full-scale development stages, and so the applicability of laboratory-scale results to full-scale design and operation is therefore limited and poorly understood. We aimed to circumvent this knowledge gap and to inform the design and interpretation of future laboratory trials using *scale-down* of an existing biotechnology, as opposed to scale-up, to investigate the impact of scale and geometry on bioreactor performance, ecology, and microbial community physiology.

**Figure 1:**
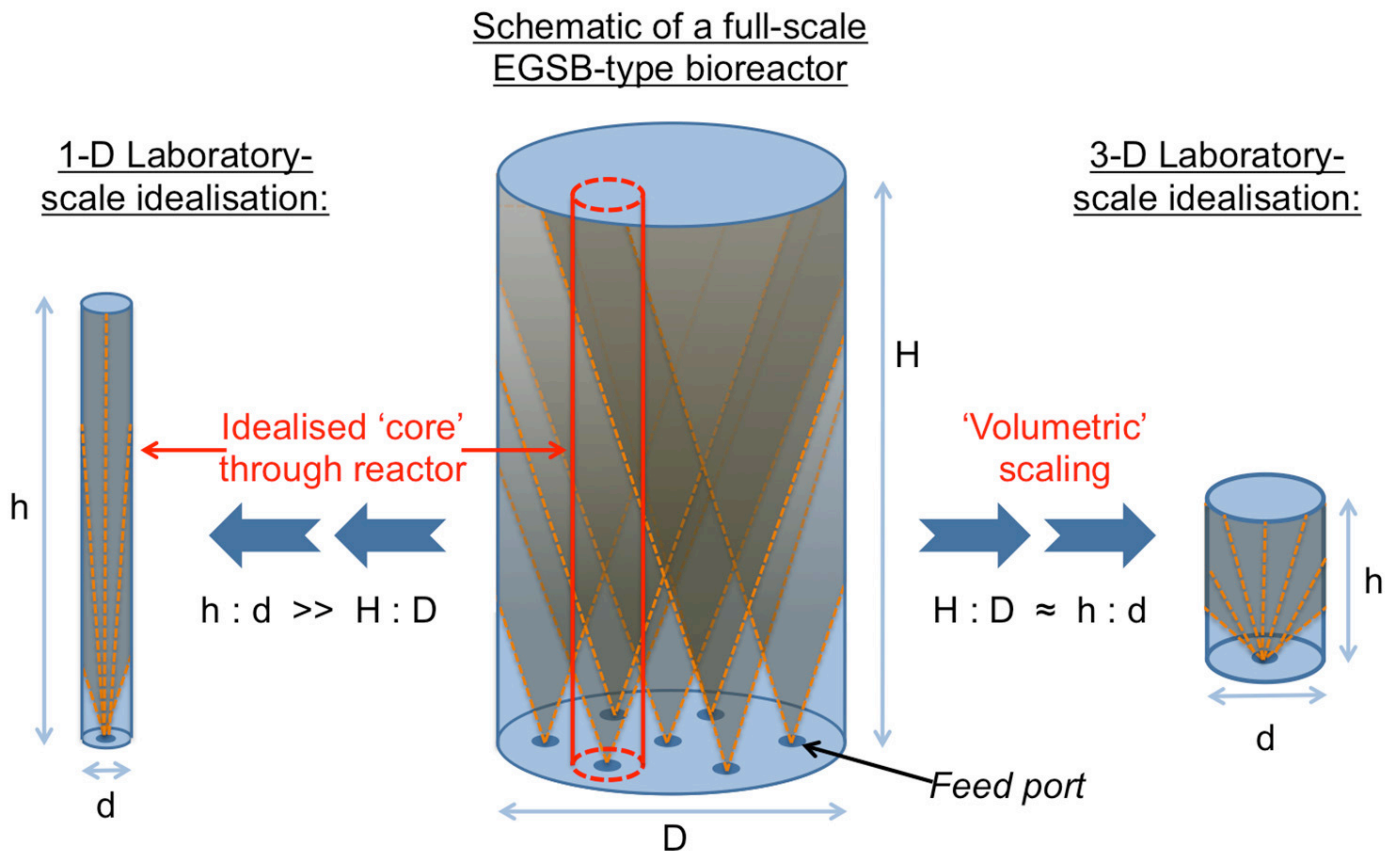
**Schematic showing scale-down logic for the design of the 1-D and 3-D laboratory-scale bioreactor geometry idealizations**.

A full-scale expanded granular sludge bed (EGSB) bioreactor operated at a Scottish whisky distillery was selected as the full-scale bioreactor for this study. The EGSB is an anaerobic digester type utilizing retained granular biomass for high-rate treatment of high-strength, low-solids industrial wastes. EGSB bioreactors have previously been used in many laboratory-scale trials investigating adaptation of the EGSB to the treatment of a growing range of wastes (Pereira et al., 2002; Fang et al., 2011), contaminants (Collins et al., 2005; Enright et al., 2005; Scully et al., 2006; Londoño and Peñuela, 2015), and operating conditions (Syutsubo et al., 2008; O'Reilly et al., 2010) which, if applicable at full-scale, have the potential to revolutionize the way we treat wastewater. Hence, this bioreactor design represents an ideal reactor type in which to investigate the effects of scale. A broad range of EGSB reactor designs (Arcand et al., 1994; Kato et al., 1994; Karnchanawong and Wachara, 2009) are commonly used in laboratory-scale studies. Recognizing that laboratory-scale reactor design is likely to influence both performance and underlying physiology and ecology, we designed two laboratory-scale bioreactor idealizations, described here as “1-D” and “3-D” bioreactors (Figure 1), to mimic the full-scale EGSB in the laboratory and to enable evaluation of scale effects. Where possible the 1-D and 3-D laboratory-scale EGSBs were operated under near-identical conditions to the full-scale bioreactor, including the use of a common inoculum and maintaining common substrate (distillery wastewater), organic loading rate (OLR), hydraulic retention time, operating temperature, and upflow velocity between scales and idealizations. We applied a range of physical and chemical monitoring techniques coupled with specific methanogenic activity assays and high-throughput 16S rRNA gene sequencing (V4 region) to evaluate differences in performance, physiology, and ecology between:

- The full- and laboratory-scale bioreactors.
- The laboratory-scale idealizations.

## Materials and methods

### Full-scale bioreactor design and operation

**Figure 2:**
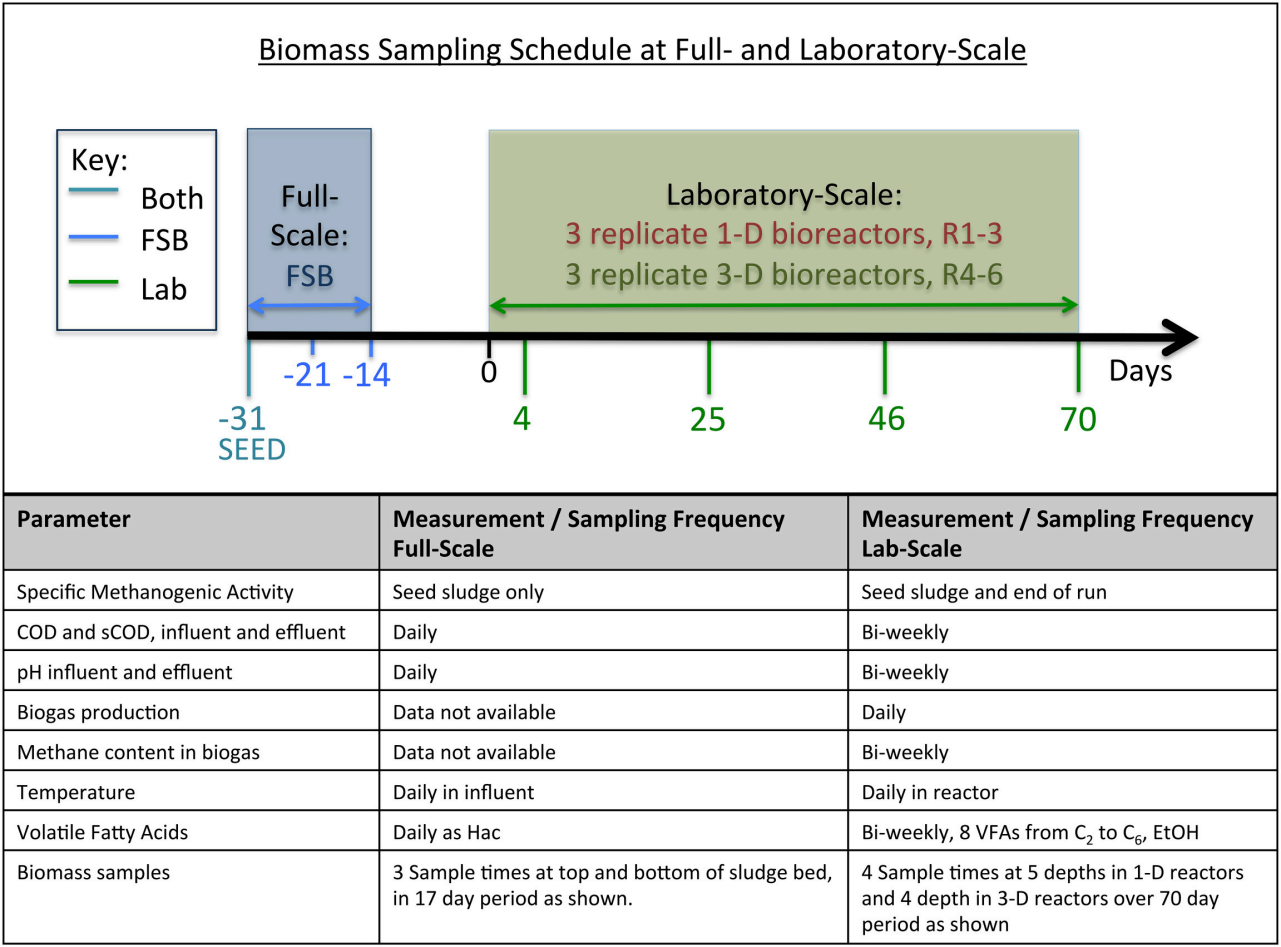
**Schematic overview of experiment showing experiment duration and relative timings of biomass sampling for full- and laboratory-scale bioreactors, and, summary of physico-chemical monitoring parameters**.

The full-scale bioreactor (FSB) is one of a set of three anaerobic digesters operated at a Scottish whisky distillery to treat spent water from the distillation of alcohol from maize. FSB is a second-generation EGSB described as the External Circulation Sludge Bed (ECSB) bioreactor (Meyer and Edwards, 2014). The primary variant between the EGSB and the ECSB is the inclusion of two gas-solid-liquid separators in the bioreactor to improve process stability and biomass retention under high OLR. Additionally, the ECSB design includes sampling ports distributed with depth in the reactor vessel enabling spatial, as well as temporal, sampling of granules from the sludge bed. The ECSB maintains the defining features of the EGSB however i.e., it is an anaerobic, upflow, retained-biomass system using liquor recycling to promote bed expansion and mixing, and treatment is underpinned by the microbial activity of the granular sludge bed. FSB has a total working volume of 425 m^3^, geometric diameter-to-height ratio of 7:12, and is operated semi-continuously at 37°C and a mean OLR of 9 g COD/L_reactor_.d (*s.d*. 2 g COD/L_reactor_.d). Operational data from FSB, obtained for a 6-month period prior to design of the laboratory-scale trials, were used to determine operating parameters for the laboratory bioreactors (Table 1). In addition to defining operating parameters, FSB served as the source of seed sludge for the laboratory-scale bioreactors, which was obtained 31 days (D-31) prior to commencement of the laboratory trial. Distillery wastewater was used as substrate at both scales. Additional biomass samples were drawn from FSB at days D-21 and D-14 to determine the structure of the microbial community at full-scale with depth and time (Figure 2). Operating and performance data reported for FSB were recorded for an 85-day period (D-85 to D0) during which FSB was temporarily shut down for a 5-day interval (D-59 to D-54) for routine maintenance of upstream reactors and was also subject to temporary shutdown immediately before and after the period of study.

**Table 1:** **Design operating parameters at laboratory-scale**.

**Parameter**	**Design value or condition**
Temperature (°C)	37
Feed type	Distillery waste transported to laboratory on weekly basis.
HRT (hours)	16
Upflow velocity (m/h)	3.5
Influent pH	6
OLR	Governed by COD of distillery waste during the trial.

### Laboratory-scale bioreactor design

Two laboratory-scale bioreactor types were designed, described here as 1-D and 3-D bioreactors (Figure 1). Each type was built and operated in triplicate: the 1-D reactor set, R1–3; and the 3-D reactor set, R4–6 (Figure 2). The laboratory-scale bioreactor designs used are intended to reflect the wide range of geometries across which the EGSB is interpreted at laboratory-scale. The 1-D bioreactor type is proportionately exaggerated in the vertical direction and may be idealized as a “core” through a full-scale bioreactor. The 3-D bioreactor, by contrast, is a more direct scaling of the volumetric dimensions of a typical full-scale bioreactor but, due to practicality of scaling, the design is simplified to include only a single feed port. The diameter-to-height aspect ratios employed were 1:15 and 1:4 for the 1-D and 3-D bioreactor types, respectively and both types were of 20-L total working volume. To facilitate spatial as well temporal biomass sampling, eight sampling ports were distributed along the length of each laboratory-scale bioreactor (from P1 at the bottom to P8 at the top), which was similar to the full-scale FSB. A single solid-liquid separator was employed in each of the 1-D and 3-D bioreactors. The separators were positioned above the sludge-bed to avoid stratification of the biomass by physical separation but below the recycle line to avoid forcible mixing of the sludge-bed by passage of granules through the recycle line.

Operation of the laboratory-scale bioreactors targeted the same mean temperature, influent, upflow velocity, hydraulic retention time (HRT), and OLR as FSB (Table 1). Each of the 20-L laboratory-scale bioreactors was seeded similarly to FSB to an initial volatile suspended solids (VSS) concentration of 12 g/L. After seeding the bioreactors and commencing feeding, biogas production was monitored over 16 days until stable rates were observed. The sampling and monitoring schedule at laboratory-scale is provided in Figure 2. The operating temperature (37°C) was controlled using external water jackets. Recirculation and feeding was applied using peristaltic pumps (Watson and Marlow 300-series). Distillery wastewater served as substrate and was transferred from the distillery to the laboratory in two 640-L intermediate bulk containers (IBCs) on a weekly basis and stored at room temperature until used. All six of the laboratory-scale bioreactors were fed from a single tank utilizing in-tank mixing to promote homogenous solids delivery to the bioreactors and to ensure replicated feeding conditions. Dissimilarly to full-scale, no shut down period was applied at laboratory-scale i.e., operation was continuous for the duration of the trial.

### Physical and chemical monitoring

At laboratory-scale, bioreactor influent and effluent was sampled twice per week for analysis of pH, total and soluble chemical oxygen demand (COD, sCOD) and volatile fatty acids (VFA). COD and sCOD was measured using the closed reflux colorimetric method (Standard Methods 5220D; APHA, 2005). Particulate COD (pCOD) was calculated as the difference between the two (COD–sCOD) to indicate solids loading. VFA (C_2_–C_6_, including iso-forms of C_4_–C_6_) and ethanol concentrations were measured using a gas chromatograph (7890A, Agilent, Palo Alto, CA) equipped with a DB-FFAP capillary column and a flame ionization detector. Biogas production was recorded on a daily basis using 10-L rubber gas-bags attached to each bioreactor to collect the biogas produced for timed periods of ~2 h. Biogas volumes were measured using a graduated gas-tight syringe to empty gasbags and biogas production rates (BPR) were calculated. Methane content in the biogas was determined on a biweekly basis, to coincide with influent and effluent monitoring, using a gas chromatograph (7890A, Agilent, Palo Alto, CA) equipped with a GS-Carbon Plot capillary column and a flame ionization detector.

At full-scale, operation and monitoring data were provided by the distillery. Parameters measured were common with laboratory-scale monitoring with two exceptions. First, at full-scale, BPR and methane content in the biogas were measured for the collective output for the three on-site digesters i.e., collected data is not specific solely to the performance of FSB. By contrast at laboratory-scale monitoring was specific to individual bioreactors and a mean for each bioreactor type is reported. Second, at full-scale a single measurement was made for total VFAs (TVFA). As such, laboratory-scale VFA data were used to calculate TVFA as an acetate-equivalent based on measurements of individual VFAs for improved comparison of data between scales.

### Specific methanogenic activity testing

Specific methanogenic activity (SMA) testing was conducted using the pressure transducer method (Colleran et al., 1992). Substrates tested were acetate, propionate, butyrate, ethanol, and hydrogen/CO_2_ and each test was conducted in triplicate at 37°C. Assays using the seed sludge at D-31 represented both the activity at full-scale and activity at laboratory-scale at the beginning of the laboratory-scale trial. SMA testing was additionally conducted on samples taken from the top and bottom of the sludge beds in the 1-D and 3-D bioreactor types at the end of the trials i.e., after microbial adaptation to reduced scale and the alternative bioreactor geometries.

### 16S rRNA library preparation

Microbial community composition in the biomass was monitored by preparation of amplicon libraries targeting the V4 hyper variable region of the 16S rRNA gene using next generation sequencing (NGS) methods and a multiplexed barcoded sample preparation approach. At laboratory-scale, biomass samples (2 ml) were drawn from each port in the sludge bed and then centrifuged to remove liquor and transferred to −20°C storage within 2 h. At full-scale, sludge samples (50 ml) taken from each port in the sludge bed were drawn to sterile, air-tight containers from which 2-ml sub-samples were taken, centrifuged and then stored at −20°C until further processing. At each scale, ports were flushed of biomass prior to capturing samples to ensure that any depth-associated effects determined were not sampling artifacts. Frozen biomass samples were thawed and subject to homogenization prior to extraction. Each DNA extraction used 0.1 g (wet weight) of biomass. DNA extraction and purification was done using the FastDNA® Spin Kit for Soils (MP Biomedical) and the FastPrep® Instrument (MP Biomedicals, Santa Ana, CA) according to the manufacturer's instructions. Extracted DNA was quantified using the Broad-Range Qubit Assay (Life Technologies) and stored at −20°C until used in NGS library preparation. NGS libraries were prepared by PCR amplification of the V4 region of the 16S rRNA gene using Golay barcoded primers (Caporaso et al., 2012), with an adaptation on the forward primer, and the KAPA HiFi HotStart PCR Kit. Our forward primer (F515: GTG**N**CAGCMGCCGCGGTAA) included an additional degeneracy for improved detection of Archaea, whilst the reverse primer, R806 (GGACTACHVGGGTWTCTAAT), was as per the Caporaso design. Efficacy of our adapted primer pair was tested *in-silico* using the Ribosomal Database Project's (RDP) Probe Match tool and indicated detection of 87.59 and 90.95% of good quality bacterial and archaeal sequences, respectively (search conditions: no mismatches, sequences should lie with the region 465–866 on the *E. coli* genome i.e., limiting the search to sequences that might contain the V4 region). By contrast the original Caporaso primer pair detected 87.53 and 54.81% of bacterial and archaeal sequences in the database, respectively using the same search conditions. Thus, our primer pair offered significant improvement for the detection of Archaea and should return a relatively representative microbial community composition with respect to relative abundance of bacterial and archaeal sequences. PCR conditions applied were: 95°C for 5 min initial denaturation; with amplification proceeding for 25 cycles of 98°C for 20 s denaturation, 60°C for 15 s annealing and 72°C for 40 s extension; followed by 72°C for 1 min final extension. Each sample was assigned a unique barcode pair and PCR was conducted in triplicate to enable a high concentration of PCR product to be obtained using a reduced number of PCR cycles. Triplicate PCR products were gel-purified and quantified prior to pooling for sequencing using the High-Sensitivity Qubit Assay (Life Technologies). Positive and negative controls for sequencing were generated using triplicate blank DNA extractions for the negative control and both skewed and evenly distributed mock communities for the positive controls. The controls were each assigned three barcode pairs to enable replicate sequencing, which was used in quality control checking. The purified barcoded PCR products were normalized to 5 ng/uL DNA and pooled for sequencing. The final pool sequenced contained samples not reported here but prepared from DNA from a similar source (laboratory-scale EGSBs treating low-strength waste) and barcoded and normalized same using the same protocol as outlined above. The concentration of the final pool was 5.6 ng/uL and comprised 249 uniquely barcoded sample libraries, of which 107 sample libraries pertain to this study. The pool was sequenced using the Illumina Miseq bench-top sequencer and V2 chemistry. Sample libraries returning fewer than 5,000 raw reads (40 samples) after sequencing were re-sequenced in a further sequencing run following the same preparation protocol but with alternative sequencing barcodes assigned to the sample. This process was repeated a third time to improve coverage of 11 of the sample libraries reported here.

### Bioinformatics

The forward and reverse reads were obtained from the sequencing center in FastQ format. Each sample library across the three sequencing runs was assigned a unique identifier and the data merged for processing as a single data set comprising 730 samples libraries of which 107 are included in this study. The positive and negative control samples were sequenced in each run. The negative control, prepared as a “blank” DNA extraction and subject to PCR as other samples, yielded no reads in any of the three runs indicating that no contamination was introduced to samples by the DNA extraction procedure used. The positive controls were processed, along with all other samples, according to the *Illumina Amplicons Processing Workflow* (http://userweb.eng.gla.ac.uk/umer.ijaz#bioinformatics), which is outlined as follows. Raw forward and reverse reads were trimmed using a sliding window approach to a minimum quality score of 20 and minimum length of 10 bp using Sickle Version 1.33 (Joshi and Fass, 2011). Trimmed paired-end reads were overlapped using PANDAseq (Masella et al., 2012) with a maximum search radius of 50 bp to form single sequences covering the V4 region. Any paired-end reads that failed to overlap were discarded. The UPARSE pipeline (Edgar, 2013) was then used to construct operational taxonomic units (OTUs, used as a proxy for species) as described in https://bitbucket.org/umerijaz/amplimock/src. The overlapped sequences from each sample were multiplexed, pooled and dereplicated, and singletons were discarded. Sequences were clustered at 97% similarity and the default setting in USEARCH, in which sequences of < 32 bp are discarded, was applied. Chimeras from abundant reads were removed *de-novo* within the UPARSE pipeline as is inherent in the “cluster_OTU” command in USEARCH. Additionally, a reference-based approach was applied to remove chimeras with lower relative abundances using a gold database (http://drive5.com/uchime/uchime_download.html) and UCHIME (Edgar et al., 2011). An OTU abundance table was then generated by matching the original barcoded overlapped reads against the cleaned consensus sequences at 97% similarity. The resultant OTU table contained re-sequenced samples as individual sample libraries e.g., the sample library for sample “S1” was represented as three “repeat” libraries S1_run1, S1_run2, S1_run3. Prior to analysis and downstream processing of the OTU table, one-way subject ANOVA (http://ww2.coastal.edu/kingw/statistics/R-tutorials/repeated.html) was used to confirm abundance profiles within these repeats were similar. Where so, the samples were collated by addition e.g., reads for OTU1 in S1_run1 were added cumulatively to reads for OTU1 in S1_run2 and to reads for OTU1 in S1_run3 to produce a single sample library “S1” containing ΣOTU1 etc. Where abundance profiles were dissimilar, the library with the highest read count of the re-run libraries was used to represent that sample. Thus, after collation of repeats, the OTU table contained a single library for each sample sequenced. At this stage a final quality check on our library preparation process, across the PCR, sequencing, and OTU clustering stages collectively, was enabled by comparison of the positive control samples against known sequences. It was determined that 95.1% (*s.d*. 0.6%) and 97.0% (*s.d*. 0.4%) of OTUs in the even and skewed mock community samples matched the predicted sequences thus confirming our procedure returned high quality data.

All OTUs (4,272 at this stage) were then taxonomically classified against the RDP database at phylum, class, order, family, and genus level, using the RDPclassifier V2.6 (Wang et al., 2007) to obtain abundance tables at each taxonomic level. To determine phylogenetic distances between the OTUs, mafft V7.040 (Katoh and Standley, 2013) was used for multi-sequence alignment of the OTUs within the dataset enabling generation of an approximately-maximum-likelihood tree using FastTree v2.1.7 (Price et al., 2010). Finally, the OTU and taxonomic abundance tables and FastTree were reduced to include only the 107 sample libraries pertaining to this study, herein referred to as “the OTU table,” “taxa tables,” and the “FastTree.”

### Qualitative and statistical data analysis methods

Statistical evaluation of difference in operation and performance data with scale and bioreactor type was conducted using one-way analysis of variance [ANOVA, aov(), R] and stated *p*-values for significance were as calculated within that function. For qualitative assessment of taxonomy across the full sample set, mean relative abundance, and standard deviation at phylum level was calculated directly from count data in the RDP classified phylum level taxa table using R-Software. To enable visualization of phylogeny amongst dominant OTUs, the FastTree was trimmed to represent the 100 most abundant OTUs using the Phyloseq package in R (McMurdie and Holmes, 2013) and the trimmed tree plotted using the web application Evolview v2 (He et al., 2016). The plotted tree was annotated in Evolview using heatmaps showing the mean relative abundance of the 100 most abundant OTUs in each reactor type (FSB, 1-D, 3-D), alongside the log transform of mean relative abundance for enhanced visualization of difference in abundance between reactor types. Non-metric multidimensional scaling (NMDS) plots were used to visualize clustering of all OTUs in samples by bioreactor type, by sampling day and by sampling depth using the Phyloseq package in R. Distances used to plot NMDS were Bray-Curtis dissimilarity calculated from OTU count data and GUnifrac distances (Chen, 2012) calculated using the FastTree and enabling inspection of grouping by abundance and phylogeny, respectively. Trends identified in the NMDS plots were assessed statistically using PERMANOVA [Adonis(), R-Vegan; Oksanen et al., 2014] and the respective distance measures (Bray-Curtis, GUnifrac), and *p*-values reported were computed within that function. Statistical difference in relative abundance with reactor type, and by sampling depth at laboratory-scale was determined by the Kruskall–Wallis test in R using log-transformed relative abundance data at phylum level. Benamini–Hochberg correction for multiple testing was applied and adjusted *p*-values were reported. Ecology indices calculated were rarefied richness, Simpson's index of diversity and Pielou's evenness index. In each case, rarefaction was applied to the full sample set to a common minimum (that of the lowest read count sample) using the rrarefy() function in R-Vegan. Variation of ecology indices with time and depth in the laboratory scale sample sets was assessed by fitting a linear model, lm() in R, and significance values reported were computed within that function. All R scripts used are maintained by the authors and all sequence data will be deposited with the European Nucleotide Archive (PRJEB18489).

## Results and discussion

### Operation at full- and laboratory-scales

Qualitatively, operation of FSB (Table 2) was variable with respect to OLR and temperature but relatively stable with respect to both total COD content and the relative proportions of soluble, and particulate, components. The variability in operation arose from a 5-day shut down period (D-59 to D-54) in which both volumetric loading rate (VLR) and temperature fluctuated. The maximum and minimum values for VLR (2.82 and 0.67 L/L_reactor_.d) and the minimum recorded temperature (30.0°C) were recorded during the re-start of operations. Neither extreme of loading rate was sustained for more than two HRTs, nor repeated during the period of interest, and temperature fluctuation was maintained for < 2 HRTs.

**Table 2:** **Temperature, loading, and influent characteristics of FSB (D-85 to D-0) and laboratory-scale bioreactors (D0–D70)**.

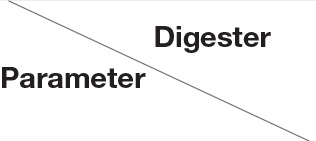	**FSB^a^**	**Laboratory-scale**^a^	
		**1-D**	**3-D**
Temperature (°C)	36.1 ± 1.0	37.1 ± 0.5	37.0 ± 1.6
VLR (L/L_reactor_.d)	1.6 ± 0.3	1.6 ± 0.1^b^	
OLR (gCOD/L_reactor_.d)	9.9 ± 3.0	9.4 ± 1.4	
pH	7.0 ± 0.2	6.2 ± 0.4	
COD (mg/L)	6530 ± 950	4710 ± 730	
sCOD (% COD)	67.2 ± 4.2	75.0 ± 10.5	
pCOD (% COD)	32.8 ± 4.2	25.0 ± 10.5	
TVFA (mg as acetate/L)	1424 ± 298	766 ± 157	
Ethanol (mg/L)	Not determined	54 ± 109	

a *Mean ± standard deviation*.

b *Both types of laboratory-scale bioreactors were fed with the same feedstock*.

Operation at laboratory-scale (Table 2) was comparatively stable yet significant difference was determined in bioreactor influent characteristics between scales. Total COD, TVFA, pH, and the proportion of pCOD in the influent were significantly lower (*p* < 0.001, ANOVA) at laboratory-scale than that at full-scale. These physico-chemical differences, each of which may impact the underlying microbial community, arose from the influence of seasonal variation in productivity at the distillery on wastewater strength and composition. Further difference in operation between scales was that no disruption to operation was applied at laboratory-scale. Together, seasonal variation of wastes and semi-continuous feeding indicate that scalability of biotechnologies must be considered in the broadest sense and not only in relation to bioreactor volume and design. Operating data at laboratory scale indicated that no significant difference (ANOVA) in operation occurred between either the 1-D and 3-D bioreactor types, nor within triplicate bioreactor sets such that the laboratory reactors may be described as true replicates in terms of operating conditions applied.

### Performance indicators at full- and laboratory-scales

The performance indicators at full-scale (Table 3) qualitatively indicate stable performance. Whilst VFA accumulation was observed in the effluent, mean COD and sCOD removal efficiencies were both high and stable. By contrast, pCOD removal was stable but low, however low pCOD removal is consistent with EGSBs reported elsewhere (Chan et al., 2009). Biogas production at full-scale was recorded for the plant as a whole i.e., a single biogas production rate was measured collectively for the three digesters on-site. It is noted however that for the period of study, mean methane content in the biogas was 70.0% (*s.d*. 2.9%) across the three on-site digesters and was relatively stable. Mean biogas production rate for FSB inferred as a proportion of the total was produced across the plant was approximately 2.5 m^3^/${\text{m}}_{\text{reactor}}^{3}$.d. Although, ethanol was not monitored for FSB, ethanol accounted for about 2% of the influent COD in the laboratory-scale bioreactors (Table 2) and was almost completely degraded (>94% on average) in the effluent (Table 3).

**Table 3:** **Effluent characteristics and performance indicators of the full- and the laboratory-scale bioreactors**.

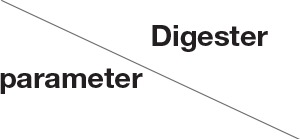	**Full-scale**	**Laboratory-scale**	
		**1-D**	**3-D**
pH	6.9 ± 1.3^a^	7.2 ± 0.2	7.3 ± 0.2
TVFA (mg as acetate/L)	282 ± 117	12.2 ± 13.4	30 ± 58
Ethanol (mg/L)	Not determined	3.1 ± 2.7	0.0 ± 0.0
COD removal (%)	70.7 ± 11.7	91.1 ± 5.1	88.1 ± 4.8
sCOD removal (%)	80.1 ± 12.6	93.7 ± 2.3	92.0 ± 4.9
pCOD removal (%)	49.8 ± 13.0	77.9 ± 28.3	69.3 ± 26.0
BPR (L/L d)	-^b^	2.95 ± 0.91	3.01 ± 0.61
CH_4_ ratio (%)	–	74.5 ± 3.5	72.8 ± 6.4

a *Mean ± standard deviation*.

b *Biogas production in the full-scale bioreactor was monitored only collectively with two other bioreactors in parallel*.

At laboratory-scale, a high degree of reproducibility was found between bioreactors in the 1-D and 3-D replicate sets with no significant difference in performance found for any indicator except for TVFA in the 1-D bioreactors (*p* < 0.05, ANOVA). As such, performance indicators at laboratory-scale are presented as a mean by bioreactor type rather than for individual bioreactors (Table 3). For each of pH, COD removal efficiency, sCOD removal efficiency, BPR and methane content in the biogas, the 1-D and 3-D bioreactor types both demonstrate stable performance throughout the trial. As with the full-scale bioreactor, pCOD removal efficiency was lower than sCOD removal efficiency but dissimilarly, pCOD removal efficiency at laboratory-scale was somewhat unstable. It was observed that the higher pCOD efficiency recorded at laboratory-scale, particularly in the 1-D bioreactors, declined over the duration of the trial suggesting that increased efficiencies recorded may be a temporal phenomenon related to the age of the bioreactors. For each of COD, sCOD, and pCOD removal, the laboratory-scale bioreactors significantly out-performed (*p* < 0.01, ANOVA) the full-scale bioreactor. Tentatively, this suggests that tightly controlled laboratory studies have the potential to exaggerate treatment efficiency as compared to more variable full-scale operation; however as influent characteristics varied between scales this is not conclusive.

No significant difference was found between the two laboratory-scale bioreactor idealizations with respect to bioreactor pH, pCOD removal efficiency, or biogas production. However, significant differences were found between each of total VFA accumulation, COD and sCOD removal efficiencies and methane content in the biogas (*p* < 0.05, ANOVA), with the 1-D bioreactors marginally out-performing the 3-D systems. As laboratory-scale operating conditions were identical, this observation indicates that laboratory idealization *does* influence performance.

### Community physiology at full- and laboratory-scales

**Figure 3:**
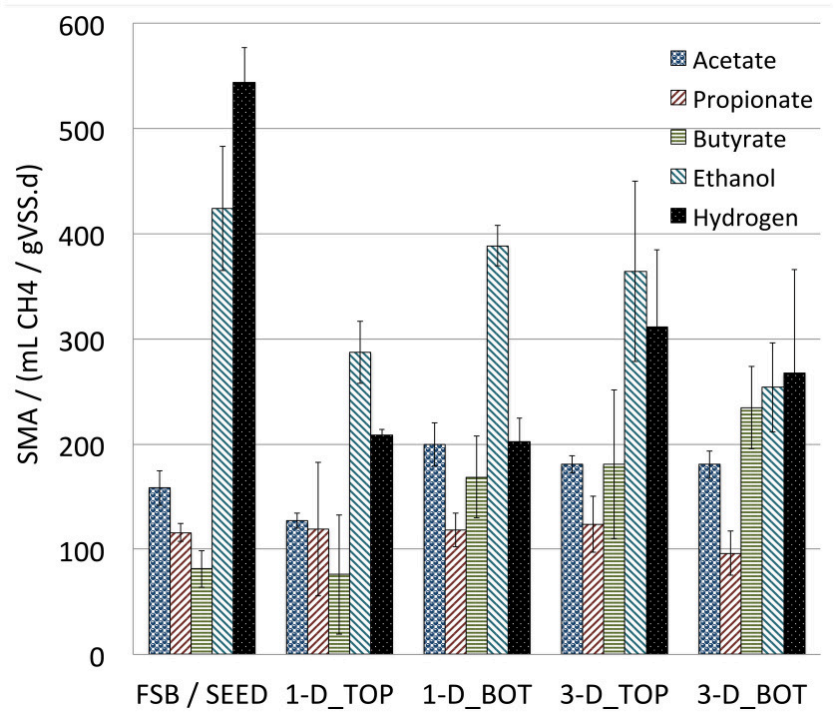
**Grouped bar plot showing SMA recorded in biomass from full-scale (SEED) and laboratory-scale bioreactors at the beginning (SEED) and end of the trial at the top (_TOP) and bottom (_BOT) of the sludge beds against specific substrates: acetate, propionate, butyrate, Ethanol, and hydrogen**. Bars show blank-adjusted mean and error bars show standard deviation of triplicate measurements.

Community physiology was investigated using SMA testing of biomass samples from the full-scale bioreactor (Port 2 on D-31, also used as seed-sludge for laboratory-scale bioreactors) and biomass samples from the top and bottom of the sludge bed in each of the 1-D and 3-D laboratory-scale bioreactor sets at the end of the trial (D70). At full-scale and in the seed sludge used at laboratory-scale (FSB/SEED Figure 3), hydrogenotrophic methanogenic activity was dominant. This was unexpected as acetoclastic methanogenesis is commonly assumed to be the dominant metabolic pathway in engineered AD systems (O'Flaherty et al., 2006). The literature has several reports of low-temperature laboratory-scale EGSB systems dominated by hydrogenotrophic methanogenesis (McKeown et al., 2009; O'Reilly et al., 2010). As the temperature in this study (37°C) was not low, it may be inferred that variable loading of higher solids wastes may induce similar stresses on acetoclastic methanogens by, for example, ammonia inhibition arising from protein degradation (Westerholm et al., 2011). By the conclusion of the laboratory trials where solids delivery was lower than at full-scale and loading was more stable, a significant decline (*p* < 0.001, ANOVA) in hydrogenotrophic activity was recorded at laboratory-scale, which in real terms was most pronounced in the 1-D bioreactor types.

Comparison of physiological profiles between biomass adapted to the 1-D and 3-D laboratory-scale bioreactor types (i.e., at D70) indicated that no significant difference in activity was found using acetate, propionate, butyrate or ethanol as specific methanogenic substrates. However, a significant difference (*p* < 0.05) was found in activity against hydrogen with biomass from the 3-D bioreactor type exhibiting a more strongly hydrogenotrophic profile than the 1-D bioreactors. As such, laboratory-scale bioreactor idealization may influence not only performance but the inferred route to methanogenesis in laboratory studies, an important finding for studies of new biotechnologies intended to inform full-scale process design.

Comparison of the spatial distribution of activity in the biomass from the two laboratory-scale reactor types indicated that the 1-D bioreactors exhibited zoned community physiology whist the 3-D bioreactors did not. In the 1-D bioreactors, activity was significantly higher in sludge from the bottom, than from the top, of the sludge bed against both acetate and ethanol (*p* < 0.001, ANOVA). By contrast, no significant difference in activity with depth was found in the 3-D type bioreactors for any substrate tested. Whilst the precise mechanisms promoting stratification of activity were not elucidated here, our data demonstrates that the depth at which biomass is sampled from 1-D type EGSB bioreactors at laboratory-scale might influence both the inferred dominant methanogenic pathway in the biomass and loading capacity of such bioreactors.

### Microbial community composition and structure at full- and laboratory-scale

#### Qualitative overview of microbial community at full- and laboratory-scales

The OTU table containing the quality filtered, chimera and singleton free reads clustered into a total of 2,929 OTUs at 97% sequence similarity for the complete data set across 107 sample libraries. Two sample libraries returned fewer than 5,000 sequences per library (both on the 1-D bioreactor R3: at port P1 on day D25 and at port P2 on day D46) and were excluded from the remaining analyses such that the lowest read count for any library was 5,232 reads. The final distribution of sample libraries per bioreactor type was: 5 samples for FSB; 53 samples across the 1-D triplicate set; and 47 samples across the 3-D triplicate set. The mean number of reads per sample library was 74,034 (*s.d*. 89,192). Rarefaction curves plotted for all samples (Figure S1) indicate that saturation was not reached for any sample sequenced. Taxonomically, OTUs identified across all samples were assigned across 24 known phyla with a mean of only 1.33% of OTUs per sample assigned as “unknown phyla” (*s.d*. 1.55%) however a mean of 36.08% of OTUs per sample were assigned as “unclassified bacteria” (*s.d*. 7.88%) and 0.75% as “unclassified archaea” (*s.d*. 0.04%). The most dominant phyla (mean relative abundance greater than 0.5%) in the sample set were *Euryarchaeota, Proteobacteria, Chloroflexi, Firmicutes, Bacteroidetes, Synergistetes*, and *Thermotogae* representing a mean relative abundance of 19.99 (*s.d*. 8.70), 13.22 (*s.d*. 3.28), 8.45 (*s.d*. 2.24), 7.70 (*s.d*. 3.63), 5.72 (*s.d*. 3.21), 4.76 (*s.d*. 2.11), and 0.50 (*s.d*. 0.19) %, respectively. Collectively, the remaining 17 phyla were attributed < 0.5% of mean relative abundance thus the data appears skewed at phylum level. At lower taxonomic levels, the OTUs were classified across 62 classes, 92 orders, 180 families, and 378 genera. Ecology indices for the sample set were calculated using the OTU table by rarefying all samples in the sample set to a common read count of 5,232 (minimum in the set). Mean rarefied richness across all samples was 460 (*s.d*. 55) OTUs, Simpson's index of diversity was 0.975 (*s.d*. 0.01) and Pielou's evenness index was 0.763 (*s.d*. 0.02). Qualitatively then the data describes a phylogenetically rich and diverse community with relatively even distribution of abundance in the microbial population across the sample set at species (OTU) level.

**Figure 4:**
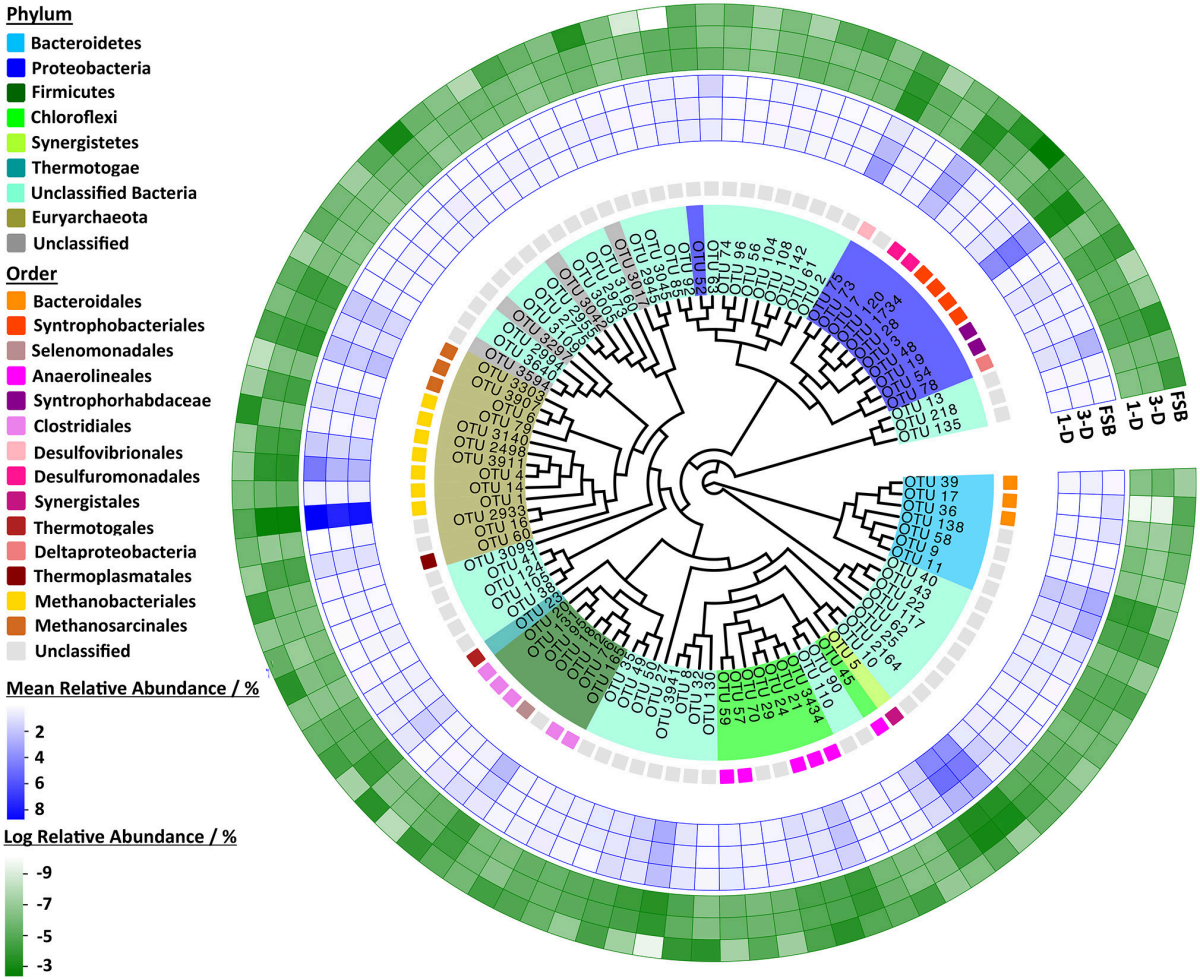
**Maximum likelihood phylogenetic tree for representative 16S rRNA gene sequences of the 100 most abundant OTUs across the sample set**. Color legends indicate OTU assignment at phylum and order level and outer rings correspond to mean relative abundance and log normalized relative abundances for each OTU in each of the 1-D, 3-D, and FSB sample sets.

Each of the 100 most abundant OTUs across the sample set (Figure 4) was present in each of the full-scale and laboratory-scale bioreactor communities indicating strong phylogenetic similarity between the communities at both scales and in each laboratory idealization. The relatively most abundant order identified was *Methanobacteriales*, accounting for seven of the 100 most abundant OTUs. Of those, six were identified at genus level as *Methanobacterium* including the most dominant OTU present (OTU_1). *Methanobacterium* are H_2_/CO_2_ and formate-utilizing methanogens (Madigan et al., 2009), so the finding was somewhat unexpected as acetate-utilizing methanogens typically dominate EGSBs. Nonetheless, this finding supports SMA data, which indicated dominance of the hydrogenotrophic pathway. The archaeal order *Methanosarcinales* was also relatively highly abundant at both scales, comprising three OTUs amongst the 100 most abundant, each of which classified as *Methanosaeta* at genus level. *Methanosaeta* are filamentous, acetoclastic methanogens associated with granule formation and maintenance and thought to form the core of anaerobic granular biofilms (Hulshoff Pol et al., 2004). Whilst inspection of mean relative abundances of the 100 most abundant OTUs in the sample set indicates that phylogeny is maintained between scales, inspection of log relative abundances indicates that there are stronger similarities between the 1-D and 3-D laboratory-scale communities than between laboratory and full-scale communities. This may reflect the differences in operating conditions applied between scales.

#### Variability in community structure and phylogeny between scales, replicate bioreactors, and with depth

**Figure 5:**
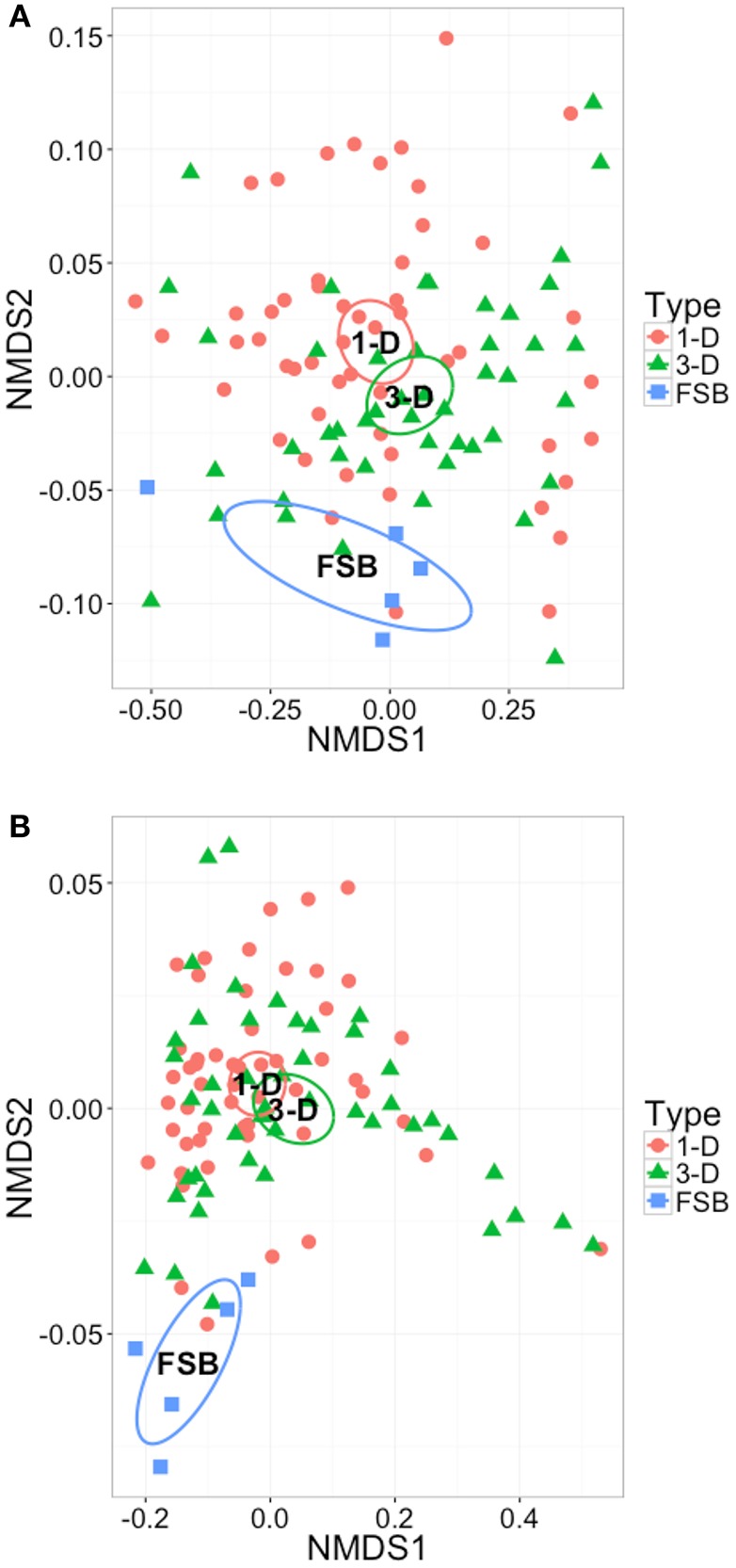
**Two-dimensional NMDS ordination plots of all OTUs in the sample set, grouped by reactor type, and plotted using (A)** structure based Bray-Curtis similarity, stress = 0.044, **(B)** phylogeny based GUniFrac distance and alpha = 0.5, stress = 0.062. In each case ellipses show 95% confidence interval of the standard error of the samples in each group around the population mean.

Ordination plots were coupled with PERMANOVA using two alternative distance metrics; Bray-Curtis and GUniFrac alpha = 0.5 to determine variability in community structure and phylogeny across all OTUs in the sample set between scales. Across all time points sampled, the laboratory-scale bioreactor communities cluster more closely to each other than to the full-scale bioreactor from which the seed sludge was drawn (Figure 5). The plot using count data (Figure 5A) clusters less distinctly than that using phylogenetic distances (Figure 5B), suggesting variability in community structure but more stable community membership at both scales. PERMANOVA was applied and determined that no significant difference in structure was found between communities from the full-scale, 3-D, or 1-D bioreactors but that significant difference was found using GUnifrac distances [*p* < 0.021, adonis()]. Thus, phylogeny appears relatively stable, yet distinct with scale, suggesting community adaptation at reduced scale did occur. The precise driver for change lacks certainty however as scale was not the only difference between the full- and laboratory bioreactors, differences in operating conditions also occurred.

A similar approach was applied to determine replication between bioreactors within 1-D and 3-D laboratory-scale test sets. No significant difference was found in species relative abundance or phylogeny across replicate 1-D bioreactors. Thus, replicated 1-D communities underpinned replicated performance metrics. However, a significant difference [*p* < 0.001 adonis(), Bray-Curtis] was found between the 3-D bioreactors estimated to account for 16% of variance across the samples despite the fact that the 3-D bioreactors appeared highly replicated with respect to operation and performance, demonstrating the importance of running replicate bioreactors during laboratory trials.

**Figure 6:**
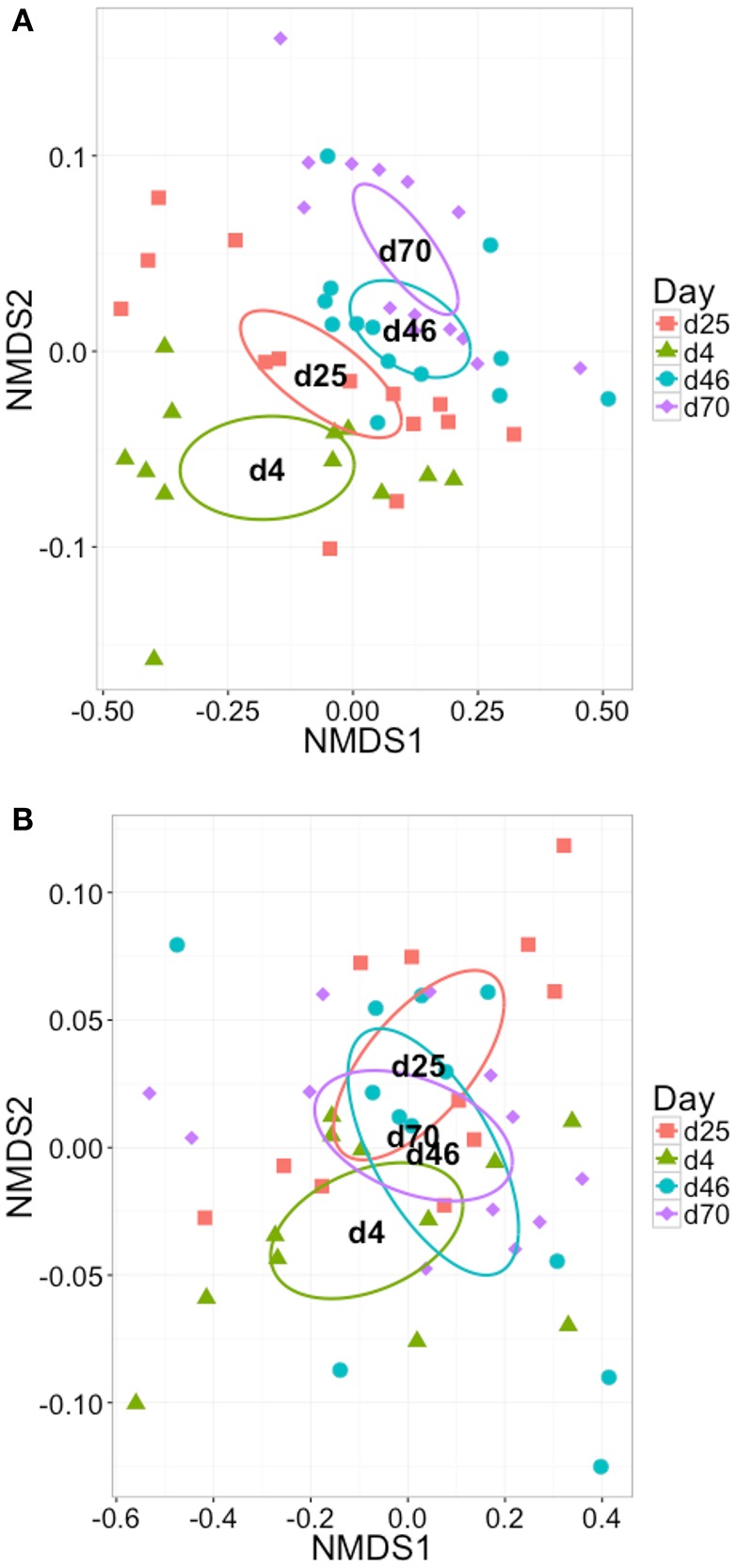
**Two-dimensional NMDS ordination plots of all OTUs in the sample set grouped by sampling day and plotted using Bray-Curtis similarity for (A)** 1-D laboratory-scale samples, stress = 0.127, and **(B)** 3-D laboratory-scale samples, stress = 0.169. In each case ellipses show 95% confidence interval of the standard error of the samples in each group around the population mean.

**Figure 7:**
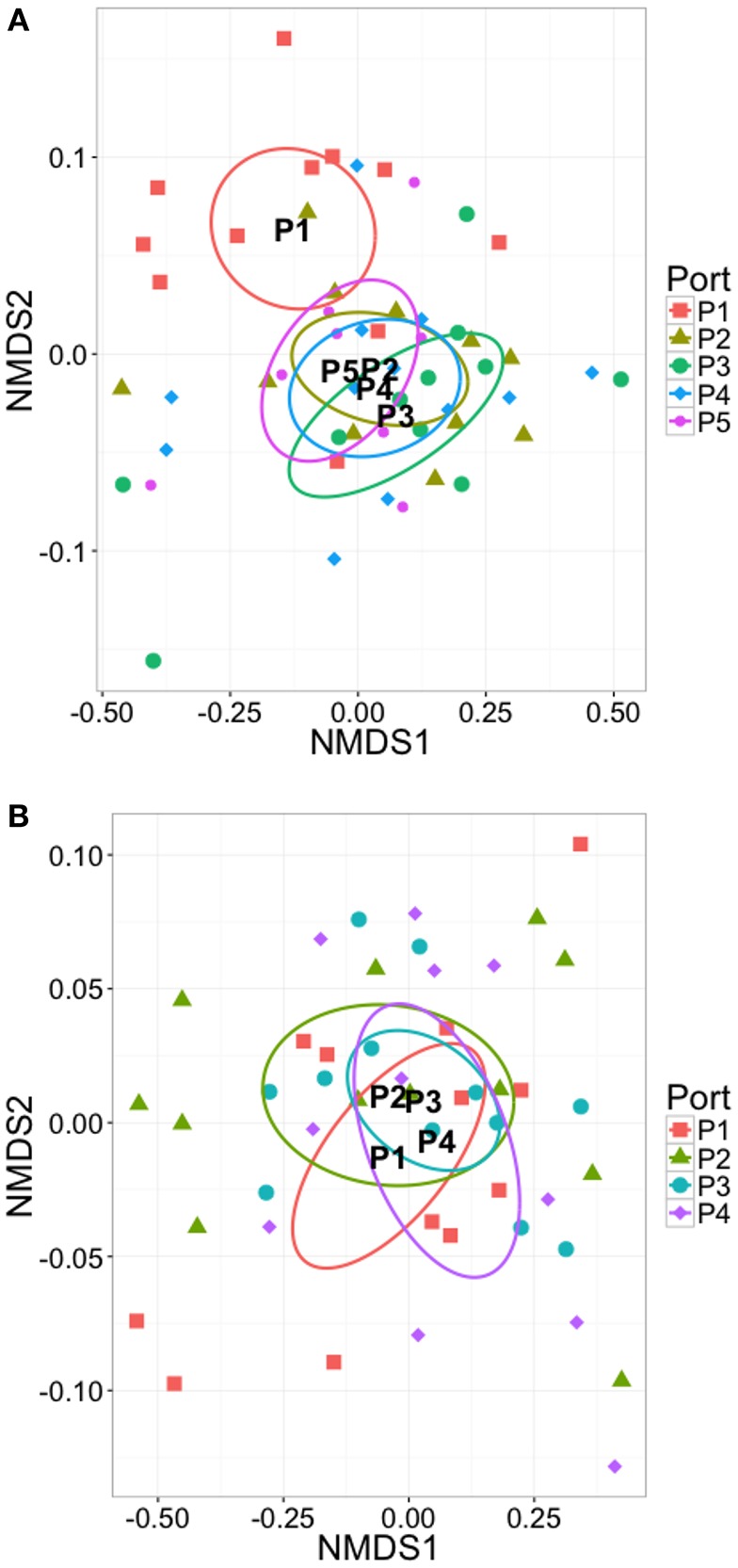
**Two-dimensional NMDS ordination plots of all OTUs in the sample set grouped by sampling depth and plotted using Bray-Curtis similarity for (A)** 1-D laboratory-scale samples, stress = 0.118, and **(B)** 3-D laboratory-scale samples, stress = 0.129. In each case ellipses show 95% confidence interval of the standard error of the samples in each group around the population mean.

Differences in microbial community structure with time (Figure 6) and depth (Figure 7) in the laboratory-scale bioreactor sets was also investigated. The full-scale digester was omitted from this analysis due to lack of replicate sampling. With time, the 1-D microbial community structure appeared to evolve more than the 3-D community. Further, with depth in the 1-D bioreactors, the microbial community at the bottom of the sludge bed (P1) clusters distinctly from those samples from higher in the sludge bed whilst little distinction with depth was observed in the 3-D bioreactors. This supports the SMA data that indicated stratification of community physiology with depth occurred in the 1-D bioreactors but not in the 3-D bioreactor type. Thus, stratified community physiology is linked with stratified community structure in the 1-D bioreactors. PERMANOVA confirmed that the trend was statistically significant, with depth estimated to account for 11.8% of variation across samples in the 1-D bioreactors [*p* < 0.01, adonis()] whilst no significant trend was found with depth in the 3-D bioreactors. This points to the importance of (i) the influence of laboratory-scale idealization on spatial and temporal community structure, and (ii) appropriate sampling strategies in AD bioreactors to adequately capture microbial community composition.

#### Key taxa associated with scale, bioreactor idealization, and depth

**Figure 8:**
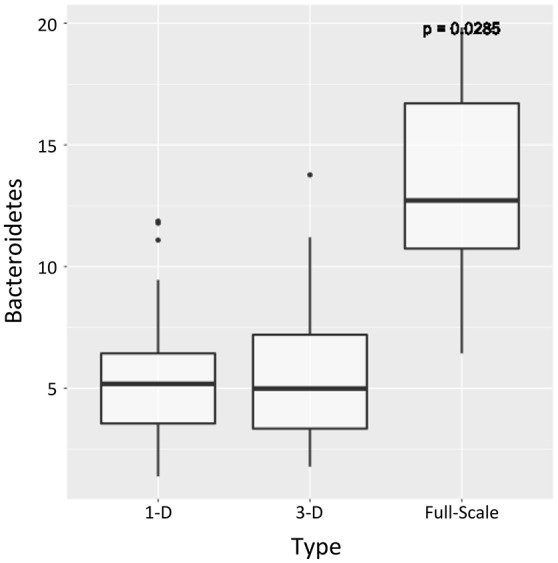
**Boxplot showing relative abundance (%) across each sample set for the phylum Bacteroidetes, the relative abundance of which was significantly greater at full-scale than at laboratory -scale (Kruskal–Wallis with Benjamini–Hochberg correction on ***p***-value, ***p*** < 0.05)**. The bands show the median value for each group; bottom and top of boxes show the first and third quartiles; and whiskers show maximum and minimum values with 1.5 of IQR of upper and lower quartiles.

The relative abundance of 5 phyla varied significantly with bioreactor type [*p* < 0.05, kruskal.test()] of which *Bacteroidetes* (Figure 8) and *Armatimonadetes* were relatively more abundant at full-scale whilst *SR1, OD1*, and *Verrucomicrobia* were relatively more abundant at laboratory-scale. Of these, only *Bacteroidetes* contributed more than 1% of the total community relative abundance at any scale. The mean relative abundance of *Bacteroidetes* in the full-scale bioreactor FSB was 13.2% (*s.d*. 5.2%) compared to a mean of only 5.34% (*s.d*. 2.59%) at laboratory-scale thus the difference is not only significant but sizeable in real terms. Organisms of the phylum *Bacteroidetes* have been identified as core to the microbial communities in full-scale anaerobic digesters (Chouari et al., 2005; Lee et al., 2008; Rivière et al., 2009) and may be associated with degradation of long chain organics such as proteins and carbohydrates (Hernon et al., 2006; Klocke et al., 2007; Thomas et al., 2011). Increased abundance at full-scale here then may reflect the higher proportion of particulate COD in the influent to FSB rather than the scale of the reactor *per se*.

**Figure 9:**
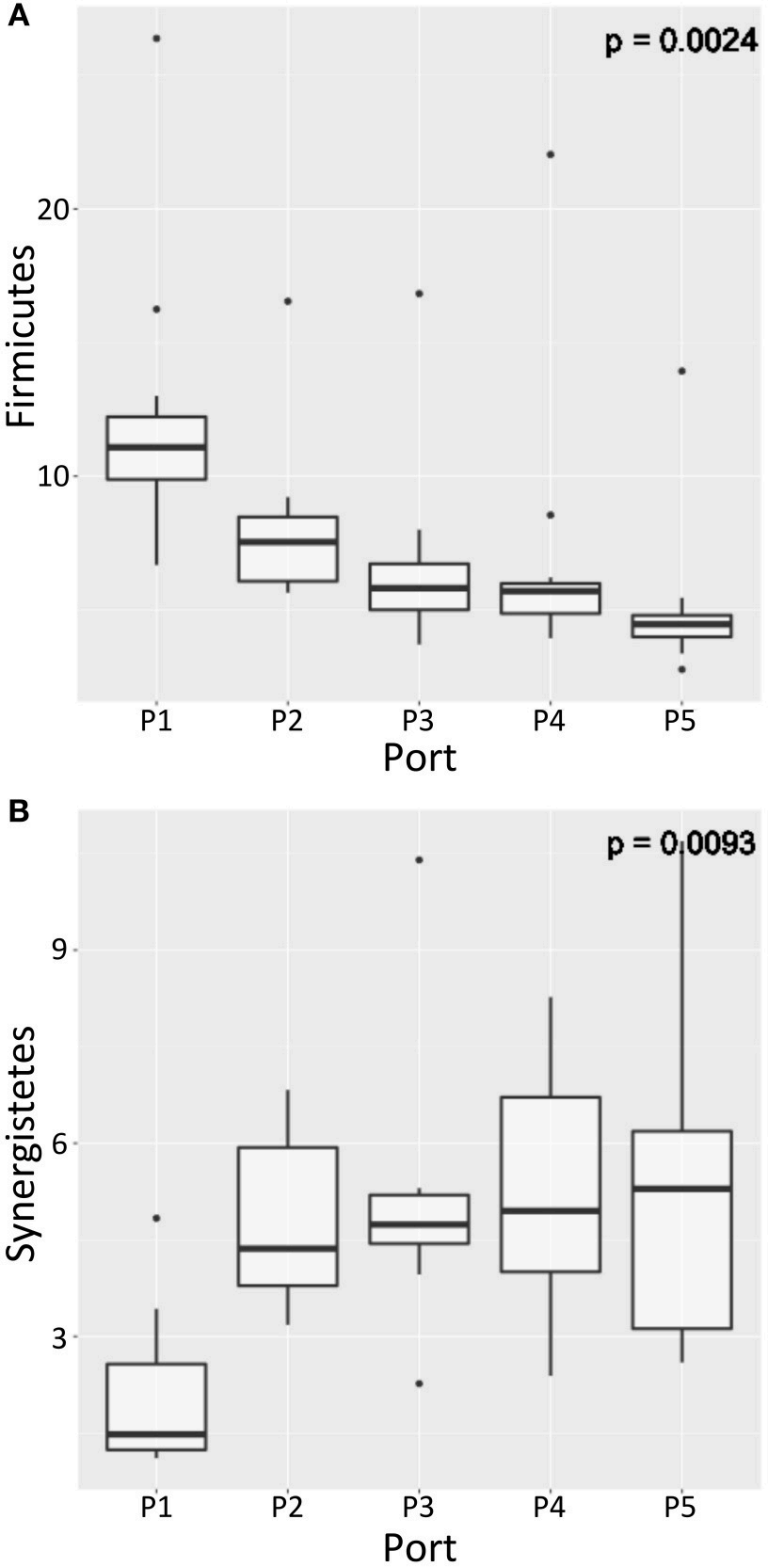
**Boxplots showing relative abundance (%) of the phyla (A)** *Firmicutes* and **(B)** *Synergistetes*, the relative abundance of which varied significantly with sampling depth (P1 at the bottom of the sludge bed to P5 at the top of the sludge bed) in the 1-D laboratory-scale bioreactors (Kruskal–Wallis with Benjamini–Hochberg correction on *p*-value, *p* < 0.05). The bands show the median value for each group; bottom and top of boxes show the first and third quartiles; and whiskers show maximum and minimum values with 1.5 of IQR of upper and lower quartiles.

Difference in relative abundance of taxa with sampling position was assessed similarly at phylum level in the laboratory-scale bioreactors however FSB was omitted from the analysis due to lack of replicate samples. In the 3-D laboratory-scale bioreactors, no significant difference in relative abundance with depth in the sludge bed was found for any phyla. By contrast in the 1-D bioreactor type, in which stratified community physiology was observed, significant difference [*p* < 0.05, kruskal.test()] in mean relative abundance with depth was observed in two dominant phyla: *Firmicutes* whose relative abundance increased with depth (Figure 9A), and *Synergistetes* whose relative abundance decreased with depth (Figure 9B). Thus, we observe that bioreactor design significantly influenced the distribution of both microbial community physiology and taxonomy in laboratory-scale EGSB type bioreactors, with 1-D bioreactors promoting stratification of each. In each case, the difference in abundance was both significant and marked in real terms. Both *Firmicutes* and *Synergistetes* are widely reported as relatively highly abundant in engineered anaerobic systems (Satoh et al., 2007; Rivière et al., 2009; Militon et al., 2015; Chen et al., 2016) and as such significant stratification observed here demonstrates the importance of spatial, as well as temporal, sampling of bioreactors at laboratory-scale when aiming to characterize microbial community composition in such systems.

#### Ecology indices with scale, bioreactor type, and depth

**Figure 10:**
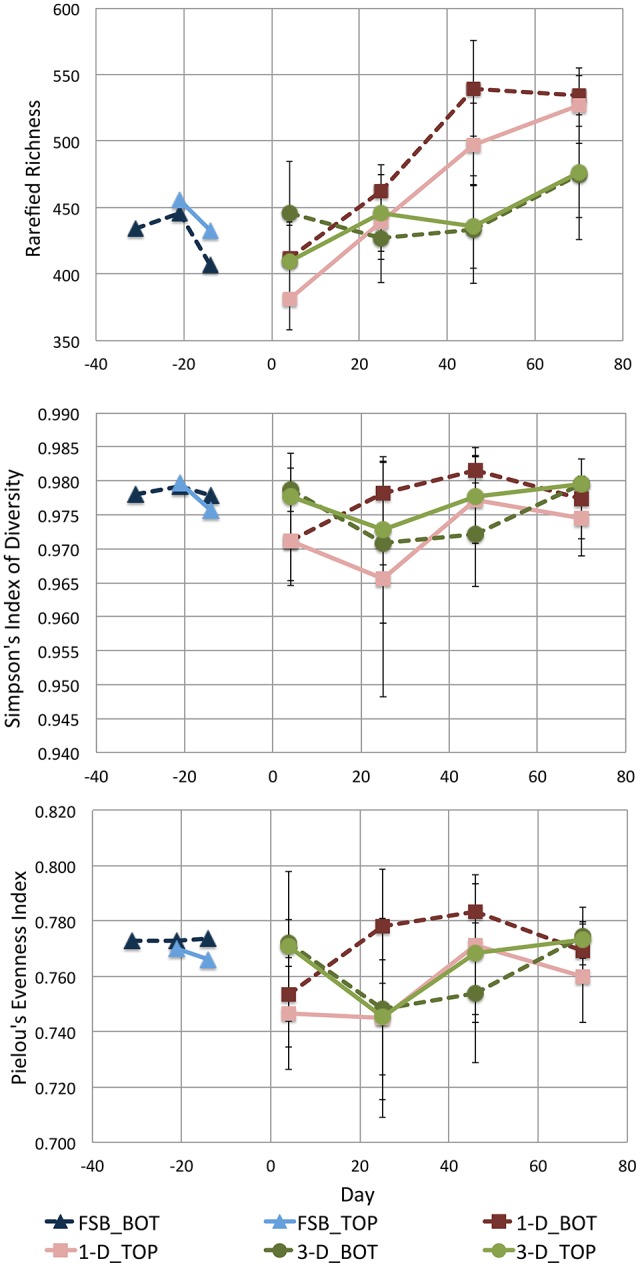
**Scatter plots showing time-series for mean rarefied richness, diversity, and evenness in each of the full-scale (FSB) and laboratory-scale (1-D and 3-D) sample sets; error bars show standard deviation**. In each case, indices are calculated using samples grouped to represent the microbial community at the top (_TOP) and bottom (_BOT) of the sludge bed in each reactor type.

Ecology indices with time and depth for each of the full-scale and laboratory-scale bioreactor sets indicate species-rich communities that are highly diverse and distributed evenly (Figure 10). Linear regression [lm(), R] determined that rarefied richness increased significantly with time in each of the 1-D and 3-D bioreactor sets (*p* < 0.001 in 1-D, *p* < 0.01 in 3-D). Increasing community richness with reduced scale was unexpected as ecological theory suggests increasing scale tends to increase richness (Brown et al., 2001). Here however scale, as in reactor volume or geometry, was not the sole difference between the laboratory- and full-scale bioreactors. Operational differences occurred too including semi-continuous feeding at full-scale and differences in physico-chemical parameters of the bioreactor influent, each of which may impact the underlying microbial community. Thus, direct attribution of the increase in richness with scale in the purest sense was not possible. Tentatively however, it could be implied that steady loading of bioreactors at reduced loading rates for both particulate COD and TVFA positively influences richness of the underlying microbial community in EGSB-type bioreactors.

The increase in richness with time in the 1-D bioreactors was more marked in real terms than in the 3-D bioreactors such that mean rarefied richness in the 1-D bioreactor set across all time points was significantly greater (*p* < 0.001, ANOVA) than that of either the 3-D bioreactors or the full-scale bioreactor FSB. Whilst direct comparison between scales was difficult, good replication of the feeding regime and influent applied between the 1-D and 3-D bioreactor types enables more direct conclusions regarding the influence of bioreactor geometry on the underlying community to be inferred at laboratory-scale. The 1-D bioreactors, in which stratification of both microbial community composition and physiology was observed, supported a more rich community in real terms than the 3-D bioreactors in which no such stratification was observed. Thus, we propose that 1-D type laboratory-scale bioreactors can support increased richness at laboratory scale by enabling a gradient of niches and microbial communities within a single reactor vessel.

## General discussion

### Disparities of scale

SMA assays indicated hydrogenotrophic methanogenesis was dominant at full-scale, which was supported by NGS data showing *Methanobacterium* was relatively the most abundant order present in the full-scale bioreactor. By contrast, hydrogenotrophic activity was diminished at laboratory-scale. We suggest that the contrast was not attributable directly to bioreactor scale but likely to the semi-continuous operation and increased solids loading applied at full-scale promoting the development of a predominantly hydrogenotrophic methanogenic system. Indeed, hydrogenotrophic methanogensis is reported elsewhere in anaerobic digester systems treating high- and very-high solids wastes (Song et al., 2010; Garcia-Peña et al., 2011; Cardinali-Rezende et al., 2012). Thus, we propose that the dominance of acetoclastic methanogenesis, as is widely reported in laboratory-scale EGSB studies, may in fact be an artifact of artificially regular feeding regimes and readily biodegradable wastes applied in laboratory trials. If laboratory-scale studies are to reflect full-scale results, laboratory operation should better reflect the variable modes of waste production, composition, and bioreactor operation likely at full-scale.

Whilst mean pCOD removal was lower at full-scale—49.8% efficiency compared with 77.9 and 69.3% in the 1-D and 3-D bioreactors, respectively—the data indicate a decreasing trend in pCOD removal efficiency at laboratory-scale. This suggests that solids gradually accumulate in “young” digesters, only to be released as the bioreactor matures. Additionally, whilst pCOD removal was lower in the full-scale digester, solids removal was more stable than in the laboratory-scale digesters as evidenced by a smaller standard deviation of pCOD removal efficiency. This suggests a better-adapted community for stable solids treatment may develop with higher solids loading, as was applied at full-scale. That *Bacteroidetes*, a bacterial phylum associated with degradation of complex organics was significantly more abundant in the full-scale digester than at laboratory-scale, appears to support this. The response of microbial communities to high-solids loading requires further study to ascertain upper limits of solids loading in EGSB type bioreactors and to better understand community adaptation.

### Influence of laboratory idealization

The design of bioreactors adopted in laboratory-scale EGSB trials is highly varied. A key aim of this study was to ascertain the influence of two distinct laboratory-scale idealizations on both bioreactor performance and the microbial community. We demonstrated that the 1-D bioreactors significantly out-performed the 3-D bioreactors with higher COD and sCOD removal efficiencies, less VFA accumulation and higher concentration of methane in the biogas produced. Further, we demonstrated that laboratory-scale idealization significantly influences both microbial community function and distribution inside bioreactors. SMA assays established that zoned community physiology developed in the 1-D bioreactors whereby biomass at the bottom of the sludge-bed was significantly more active against both acetate and ethanol than biomass from the top of the bed. Zoned physiology in the 1-D bioreactor types was underpinned zoned microbial community composition. We found distinct clustering of OTUs with depth in the 1-D digesters and 11.8% of variance in the 1-D digester community was attributed to sampling depth. Further, the relative abundance of dominant phyla *Firmicutes* and *Synergistetes* varied significantly with depth in the 1-D digester. Of those, *Firmicutes* appeared most abundant at the bottom of the 1-D type bioreactors where growth could be linked to the acetotrophic metabolism observed there. Nothing similar was found in the 3-D bioreactors. Whilst the observation that activity in 1-D type EGSB bioreactors may be zoned has been reported previously (Arcand et al., 1994), this study is novel in demonstrating that this is underpinned by stratified microbial community composition and that bioreactor geometry appears to act as a driver for stratification. Further, the study is novel in identifying that stratification in the underlying microbial community in 1-D type bioreactors appears to support increased species richness that may be associated with improved treatment observed in this bioreactor type. Whilst the precise mechanism promoting stratification in 1-D bioreactors was not elucidated here, tentatively we propose that the stratification may be driven by a confining effect of the 1-D reactor geometry on the granular sludge bed, enabling establishment of niche environments in the bioreactor.

## Conclusions and recommendations

Laboratory-scale trials typically strive to attain “steady-state” operation, but full-scale bioreactor operation may be highly variable with respect to substrate composition, strength and loading rate, and feeding and heating regimes and schedules. Here it is proposed that variable operation was the key driver accounting for differing performance and ecology between scales. Hydrogenotrophic methanogenesis dominated the full-scale bioreactor, whereas balanced acetotrophic and hydrogenotrophic methanogenesis developed in more stably operated laboratory-scale bioreactors. As such it is recommended that the variable modes of waste generation, and of bioreactor operation, should be incorporated into controlled laboratory-scale trials where subsequent scale-up is intended. Demonstrating that the apparent dominance of the acetoclastic pathway in anaerobic digestion may, in fact, be an artifact of experimental design at laboratory-scale, could lead to great opportunities for improving full-scale digester design and operation.

Laboratory-scale idealization strongly influenced each of bioreactor performance, and microbial community structure, and spatial distribution. Thus, the influence of laboratory-scale bioreactor design on the success of scale-up must be better understood. The findings underscore the importance of sufficient biomass sampling to develop time series studies and to determine spatial distribution in communities that might be wrongly assumed to be homogeneous.

## Author contributions

SC, SS, GC, and WS designed the study. SC, SS, and RD operated and monitored the laboratory-scale bioreactors. SC and SS prepared the sequencing libraries. CQ designed the primer adaptation. SC, CQ, and UI wrote the scripts for data analysis. Data analysis was conducted by SC. The results were interpreted by SC, SS, GC, and WS. SC drafted the paper and SS, GC, WS, UI, RD, and CQ revised the document. All authors approve the paper and agree for accountability of the work therein.

### Conflict of interest statement

The authors declare that the research was conducted in the absence of any commercial or financial relationships that could be construed as a potential conflict of interest.

## Supplementary material

Figure S1 **Rarefaction curves [rarefy(), R Vegan] for all samples in the sample set grouped by bioreactor type: 1-D laboratory-scale (red), 3-D laboratory-scale (green), and full-scale FSB (blue)**. Curves show saturation is not reached for any sample sequenced.
