# Supplementary material for: Bioreactor Scalability: Laboratory-Scale Bioreactor Design Influences Performance, Ecology, and Community Physiology in Expanded Granular Sludge Bed Bioreactors
Source: Front Microbiol. 2017 May 1;8:664. doi: 10.3389/fmicb.2017.00664 (PMC5410579; doi:10.3389/fmicb.2017.00664)
Supplement: Figure S1 — Rarefaction curves [rarefy(), R Vegan] for all samples in the sample set grouped by bioreactor type: 1-D laboratory-scale (red), 3-D laboratory-scale (green), and full-scale FSB (blue). Curves show saturation is not reached for any sample sequenced. [file Image1.pdf]

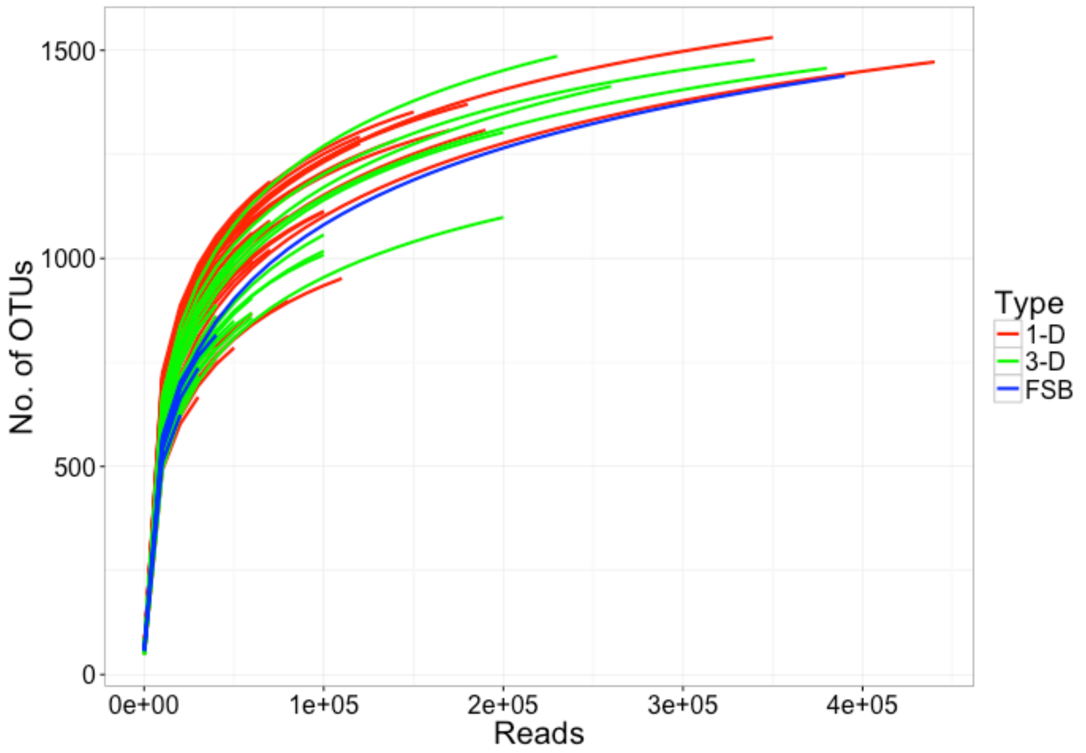

S1: *Rarefaction curves (rarefy(), R Vegan) for all samples in the sample set grouped by bioreactor type: 1-D laboratory-scale (red), 3-D laboratory-scale (green) and full-scale FSB (blue). Curves show saturation is not reached for any sample sequenced.*
